# 5-Hydroxymethylcytosine is associated with enhancers and gene bodies in human embryonic stem cells

**DOI:** 10.1186/gb-2011-12-6-r54

**Published:** 2011-06-20

**Authors:** Hume Stroud, Suhua Feng, Shannon Morey Kinney, Sriharsa Pradhan, Steven E Jacobsen

**Affiliations:** 1 Department of Molecular, Cell and Developmental Biology, University of California, Los Angeles, Los Angeles, CA 90095, USA; 2 Howard Hughes Medical Institute, University of California, Los Angeles, Los Angeles, CA 90095, USA; 3 New England Biolabs, Inc., 240 County Road, Ipswich, MA 01938, USA

## Abstract

**Background:**

5-Hydroxymethylcytosine (5hmC) was recently found to be abundantly present in certain cell types, including embryonic stem cells. There is growing evidence that TET proteins, which convert 5-methylcytosine (5mC) to 5hmC, play important biological roles. To further understand the function of 5hmC, an analysis of the genome-wide localization of this mark is required.

**Results:**

Here, we have generated a genome-wide map of 5hmC in human embryonic stem cells by hmeDIP-seq, in which hydroxymethyl-DNA immunoprecipitation is followed by massively parallel sequencing. We found that 5hmC is enriched in enhancers as well as in gene bodies, suggesting a potential role for 5hmC in gene regulation. Consistent with localization of 5hmC at enhancers, 5hmC was significantly enriched in histone modifications associated with enhancers, such as H3K4me1 and H3K27ac. 5hmC was also enriched in other protein-DNA interaction sites, such as OCT4 and NANOG binding sites. Furthermore, we found that 5hmC regions tend to have an excess of G over C on one strand of DNA.

**Conclusions:**

Our findings suggest that 5hmC may be targeted to certain genomic regions based both on gene expression and sequence composition.

## Background

Cytosine DNA methylation (5-methylcytosine (5mC)) is an epigenetic mark that is widespread in both animals and plants, and appears to play important roles in various biological processes, such as gene silencing and imprinting. Recently, studies have shown that embryonic stem cells (ESCs) and Purkinje neurons contain high levels of 5-hydroxymethylcytosine (5hmC) [1,2]. Human TET1, a 2-oxoglutarate- and Fe(II)-dependent enzyme, has been shown to catalyze the conversion of 5mC to 5hmC both *in vitro *and *in vivo *[1]. Subsequently, all mouse Tet proteins, Tet1, Tet2 and Tet3, were shown to be able to convert 5mC to 5hmC [3]. Disruption in human TET1 and TET2 is associated with diseases such as MLL-associated leukemia [4] and myeloproliferative disorders [5]. Studies have suggested that 5hmC inhibits the methyl-CpG-binding protein MeCP2 from binding DNA [6]. In addition to the exclusion of methyl-CpG-binding proteins, 5hmC may recruit unknown 5hmC binding protein(s). Moreover, because the DNA methyltransferase DNMT1 binds poorly to 5hmC [1,7], it is possible that 5hmC plays a role in excluding DNMT1 from methylating cytosines and thus may promote DNA demethylation. Importantly, 5hmC diminishes as embryonic stem cells (ESCs) differentiate, suggesting that 5hmC may play specific roles in ESCs. Indeed, mouse Tet1 has been shown to be required for ESC maintenance [3]. The function of 5hmC in mammals remains poorly understood. To further understand the role of 5hmC, it is necessary to understand where 5hmC localizes in the genome. Very recently, a genome-wide map of 5hmC was reported in mouse cerebellum [8]. 5hmC was chemically tagged and affinity enriched, and the purified DNA was sequenced. The authors found that 5hmC is enriched over genes and is positively correlated with expression levels [8].

Recently, commercial antibodies specific to 5hmC have become available. While these antibodies specifically recognize 5hmC, it is important to note that they tend to prefer densely 5-hydroxymethylated sites to single 5hmC sites (Figure S1 in Additional file 1). Here we generated genome-wide maps of 5hmC in human ESCs (hESCs) by performing hydroxymethyl-DNA immunoprecipitation followed by massively parallel sequencing with an Illumina Genome Analyzer (hmeDIP-seq). As did Song *et al*. [8], we found that a large fraction of 5hmC peaks were enriched over genes. However, we also found that 5hmC is enriched over predicted hESC enhancers, further suggesting a potential role of 5hmC in gene regulation. Moreover, we observed enrichment of 5hmC peaks with transcription binding sites such as those of pluripotency factors OCT4 and NANOG. In addition, we found that 5hmC regions correspond to genomic regions that are GC-skewed.

## Results and discussion

### 5hmC is enriched over genic regions

**Figure 1:**
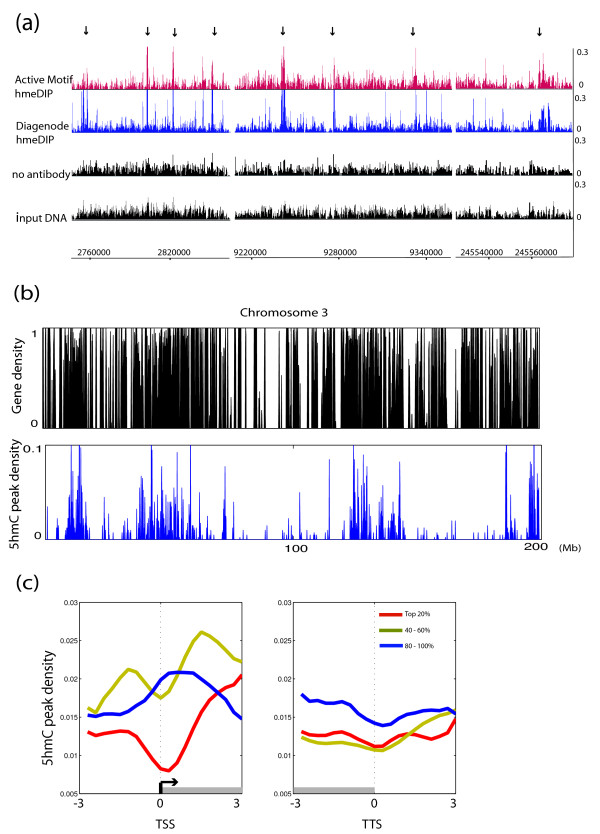
**High resolution map of hydroxymethylcytosine in human embryonic stem cells**. **(a) **Genome-browser view of hmeDIP-seq data. Two hmeDIP-seq datasets along with input DNA and 'no antibody' controls are shown. Each track is represented as normalized density of reads (reads/bp/million uniquely mapping reads). **(b) **Gene density (genes/bp) and 5hmC peak density (peaks/bp) in chromosome 3. **(c) **5hmC over genes with different expression levels. 5hmC peak density was plotted over RefSeq genes in 300-bp bins. Plots were smoothed by taking the moving average over ± 2 bins. Published RNA sequencing data [9] were used to rank the genes. TSS, transcription start site; TTS, transcription termination site.

To generate genome-wide maps of 5hmC, we performed two hmeDIP-seq experiments using two different commercial antibodies (Active Motif and Diagenode). HmeDIP-seq experiments generated 10 to 30 million reads that uniquely mapped to the human genome (Figure 1a). We defined regions for both maps by using both input DNA and 'no antibody' sequencing reads as background controls (see Materials and methods). We selected 15,324 regions that were called significant in both hmeDIP-seq experiments. The average length of the defined regions was 1.5 kb, and consistent with previous findings with chemically labeling methods [8], sex chromosomes were depleted in 5hmC regions (Figure S2a-c in Additional file 1).

The chromosomal distribution of 5hmC regions suggested that 5hmC is within gene-rich chromosomal domains (Figure 1b). Indeed, 46.2% of defined 5hmC regions overlapped with RefSeq annotated genes, suggesting a potential role of 5hmC in gene regulation. Plotting 5hmC peaks over RefSeq genes, we found that 5hmC tends to localize to transcribed regions (bodies) of genes in addition to immediate upstream regions (Figure S3a in Additional file 1). The distribution of expression levels of genes with 5hmC peaks was similar to levels of all genes, suggesting that 5hmC may not linearly correlate with expression levels (Figure S3b in Additional file 1). Plotting the distribution of 5hmC peaks over RefSeq genes with different expression levels, we observed that 5hmC is enriched near the transcription start sites of lowly expressed genes, whereas 5hmC is depleted at transcription start sites of highly expressed genes (Figure 1c). This is in contrast to data reported by Song *et al*. [8] that suggested that 5hmC levels positively correlate with expression in mouse cerebellum, suggesting possible differences in the role of 5hmC in different tissues.

### 5hmC is enriched over enhancers

**Figure 2:**
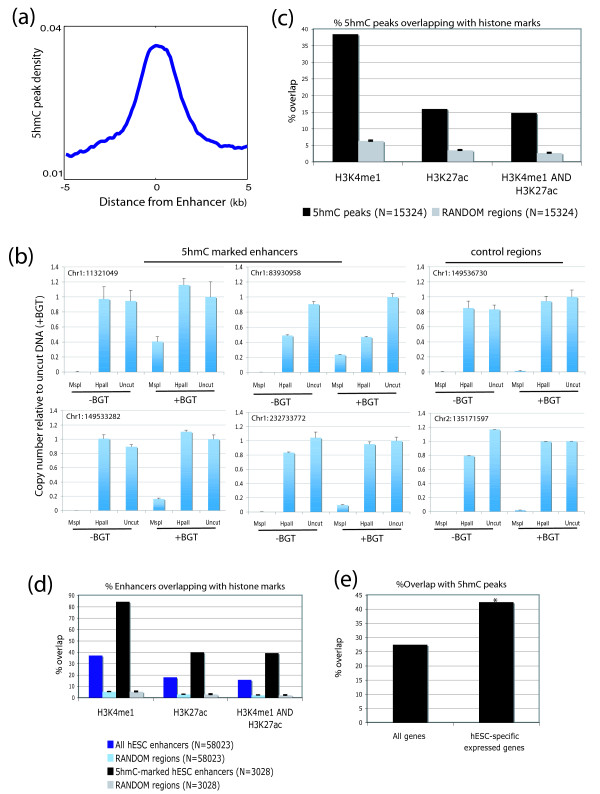
**Co-localization of 5hmC with enhancers**. **(a) **5hmC over enhancers in hESCs. 5hmC peak density was plotted over predicted hESC enhancers [9,21] in 100-bp windows. **(b) **Verfication of 5hmC at enhancers by measuring 5hmC levels at CCGG sites. Four 5hmC peaks at enhancers and two control regions were tested. MspI cannot cut glucosylated-5hmC but is able to cut 5hmC; therefore, copy numbers of MspI + beta-glucosyltransferase (BGT) represent 5hmC levels. On the other hand, HpaII can only cut unmodified DNA; therefore, copy numbers represent 5hmC + 5mC levels. Background signal was subtracted from the copy number of each sample and then normalized to the undigested glucosylated sample. Genomic locations of tested cytosines are indicated. Error bars represent the standard deviation. **(c) **Histone modifications over 5hmC peaks. The overlap of 5hmC peaks with previously defined H3K4me1- and H3K27ac-enriched regions [12] was calculated. Random regions with the same number and size distribution as 5hmC peaks were generated and overlap with histone modifications was calculated 100 times. Error bars represent standard deviation. **(d) **Histone modifications over 5hmC-marked enhancers. Predicted enhancers that overlapped with 5hmC peaks were selected. The overlap of these 5hmC-marked enhancers, as well as all predicted enhancers, with previously defined H3K4me1 and H3K27ac enriched regions [12] was calculated. Random regions with the same number and size distribution as the enhancers were generated and overlap with histone modifications was calculated 100 times. Error bars represent standard deviation. **(e) **hESC-specific expressed genes significantly overlap with 5hmC peaks. hESC-specific genes were defined as genes that were expressed in hESCs (reads per kilobase of exon model per million mapped reads (RPKM) ≥ 0.5) and silent in IMR90 cells (RPKM = 0) using published RNA-seq data [9]. *P < 0.0001.

Because a large proportion of 5hmC peaks did not fall into genic regions, we examined whether 5hmC co-localized with predicted enhancers in hESCs [9]. Indeed, we found that 5hmC peaks were highly enriched over enhancers (Figure 2a), and the magnitude of enrichment was greater than that observed over genes. We found that 3,028 enhancers overlapped with 5hmC peaks. 5hmC enrichment at enhancers was verified using a method that can measure 5hmC levels at CCGG sites by glucosylating 5hmC and digesting with the MspI restriction enzyme (MspI can digest 5hmC, but not glucosylated 5hmC) [10] (Figure 2b). Quantitative PCR assays across selected CCGG sites suggested that 5hmC levels at tested 5hmC peaks ranged from 9.6% to 36.4%, whereas at control regions the levels ranged from 0.5% to 2.7%. Enhancers are marked by chromatin signatures such as histone H3 lysine 4 monomethylation (H3K4me1) and histone H3 lysine 27 acetylation (H3K27ac) [11]. We confirmed that 5hmC peaks significantly overlapped with defined H3K4me1 and H3K27ac regions from a separate study [12] (Figure 2c), and 5hmC-marked enhancers overlapped with both of these marks to a greater extent compared to all predicted enhancers (Figure 2d). Remarkably, we observed positive correlations between 5hmC and H3K4me1 and H3K27ac despite comparing data from different cell lines. We next tested whether 5hmC marks active or poised enhancers [12], and found that 5hmC is more likely associated with active enhancers (Figure S4a, b in Additional file 1). Because enhancers regulate gene expression in a cell-type-specific manner [11], we sought to test whether genes specifically expressed in hESCs are associated with 5hmC. We defined the genes closest to each given 5hmC peak (measured from transcription start sites; maximum distance allowed = 100 kb) as 5hmC-regulated genes. We next defined hESC-specific expressed genes by using a published RNA-seq data set (H1 hESCs versus IMR-90 fibroblast cells) [9] and only selecting genes that were expressed in hESCs (reads per kilobase of exon model per million mapped reads (RPKM) ≥ 0.5) and silent in IMR-90 cells (RPKM = 0). Interestingly, we found that 42.5% of hESC-specific expressed genes overlapped with 5hmC peaks, whereas 27.5% of all genes overlapped with 5hmC peaks (*P *< 0.0001; Figure 2e). Hence, in hESCs, 5hmC shows some preference for genes specifically expressed in hESCs. This is consistent with results from previous studies suggesting that predicted enhancers from a particular cell-type mark cell-specific expressed genes [11].

To examine whether 5hmC peaks are associated with genes with specific functions, we performed gene ontology analyses using GREAT [13], which enables functional analysis of *cis*-regulatory regions such as enhancers. Interestingly, 5hmC-associated genes tended to function in processes such as embryonic pattern specification, cerebellum morphogenesis, and other developmental processes (Figure S5 in Additional file 1).

### 5hmC is enriched over transcription factor binding sites

**Figure 3:**
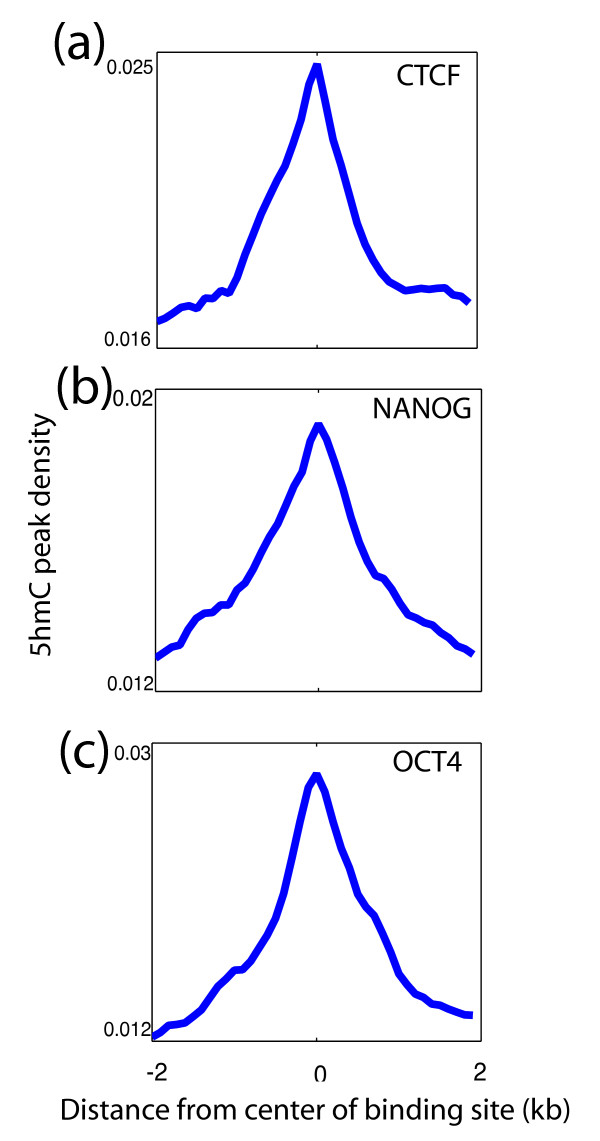
**Co-localization of 5hmC with transcription factor binding sites**. **(a) **5hmC regions over CTCF binding sites in hESCs. 5hmC peak density was plotted over the ± 2 kb relative to the center of defined binding sites in 100-bp windows. **(b) **Over NANOG binding sites in hESCs. **(c) **Over OCT4 binding sites in hESCs.

Because predicted enhancers of ESCs are enriched in some known ESC-specific transcription factors [11], we next examined the overlap of 5hmC with previously identified transcription factor binding sites (TFBSs) in hESCs [14]. Interestingly, we found that 5hmC regions were enriched over pluripotency factors NANOG and OCT4 binding sites, as well as the sites of insulator binding protein CTCF (Figure 3a-c). This result, along with the fact that 5hmC is enriched over enhancers, suggests that 5hmC may mark protein-DNA interaction sites. Visual inspection of certain hESC-specific genes [15] further confirmed overlaps between 5hmC and loci such as enhancers and TFBSs (Figure S6a,b in Additional file 1). Bisulfite sequencing and methylation-sensitive restriction digestion methods, both techniques that cannot distinguish 5mC and 5hmC, have suggested that DNA methylation levels are reduced at TFBSs and enhancers [9,12]. Because we observed enrichment of 5hmC at these sites, it is likely that the low levels of DNA methylation observed by the previous studies are at least in part 5hmC. This suggests that 5mC levels may be even lower than previously measured by bisulfite sequencing and methylation-sensitive restriction digestion methods. It is plausible to hypothesize that 5mC blocks enhancer proteins and transcription factors from binding DNA. On the contrary, 5hmC inhibits both the DNA methyltransferase DNMT1 and the methyl-CpG-binding protein MeCP2 from binding [1,6,7]. Hence, 5hmC may function in negatively regulating 5mC levels at certain protein-DNA interaction sites in order to allow protein-DNA binding of enhancer proteins and transcription factors.

### 5hmC regions are GC-skewed

**Figure 4:**
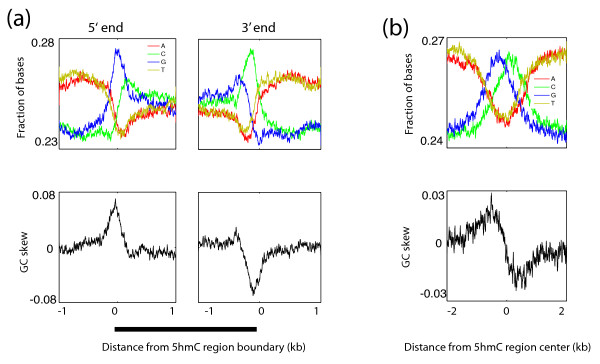
**5hmC regions are GC-skewed**. **(a) **Base composition of the Watson strand over the 5' and 3' boundaries of 5hmC regions. GC-skew = absolute value (G - C)/(G + C). **(b) **Base composition of the Watson strand over the centers of 5hmC regions.

We next examined the sequence contexts associated with 5hmC. By plotting the frequencies of nucleotides, we observed that 5hmC regions are enriched in GC content (Figure 4a, b). Furthermore, curiously, we found that 5hmC regions are GC-skewed, where Gs are enriched over Cs in the 5' ends of the regions, whereas the 3' ends of the regions had the opposite skew, where Cs are enriched over Gs (Figure 4a, b). The GC-skew was observed in 5hmC regions overlapping with genes and enhancers as well as 5hmC regions not overlapping with these elements, suggesting that this sequence composition is a common feature of all 5hmC regions (Figure S7a-c in Additional file 1). Hence, 5hmC may be targeted to GC skew regions. Because changes in the sign of GC-skew (from G rich to C rich) is thought to occur at sites of replication termination [16-18] as well as recombination hotspots [19], a speculation is that 5hmC may also mark termination sites of DNA replication or sites of recombination. With the same reasoning that 5hmC may mark sites of protein-DNA binding, such as enhancers and transcription factors, an attractive hypothesis is that 5hmC may allow binding of factors of replication or recombination.

## Conclusions

We have generated the first genome-wide map of 5hmC in hESCs, and have found that 5hmC localizes to enhancers and gene bodies. 5hmC also tended to localize to other protein-DNA interaction sites such as TFBSs, suggesting a role of 5hmC in gene regulation. Finally, we found a novel characteristic of the DNA sequences associated with 5hmC peaks, GC-skew, which suggests the possibility that sequence composition may be a signal for the deposition of this epigenetic mark.

## Materials and methods

### Hydroxymethyl-DNA immunoprecipitation and Illumina library generation/sequencing

hmeDIP experiments were performed on HSF1 hESCs as previously described [20] using commercial antibodies specific to 5hmC, except that Illumina adapter ligated DNA fragments were used as the input for the immunoprecipitation. Two experiments, one using rabbit polyclonal antibody (Active Motif, Carlsbad, CA, USA) and the other using mouse monoclonal antibody (Diagenode, Sparta, NJ, USA), were performed using 5 μg per immunoprecipitation. Input genomic DNA and no antibody controls were also kept for sequencing. Illumina libraries were generated and sequenced on an Illumina Genome Analyzer per the manufacturer's instructions.

### Data processing and analysis

Sequenced reads were base-called using the standard Illumina software. Reads were trimmed down to 50 bases due to low quality base calls in the 3' end of reads, and aligned to hg18 with Bowtie (v.0.12.4) allowing up to three mismatches. Only uniquely mapping reads were kept, and identical reads were collapsed to one read. Because the reads represent the ends of DNA libraries, for the downstream analyses, the reads were extended to represent the average fragment size of the libraries. All sequencing data have been deposited in Gene Expression Omnibus [GEO:GSE27627]. Regions were defined by using SICER (v.1.03). Only regions that were called by using both input and 'no antibody' as a background control with Benjamini corrected false discovery rate < 0.05 were kept. Finally, only regions called in both antibody hmeDIP-seq experiments were kept and analyzed. Gene ontology analysis was performed using the Genomic Regions Enrichment of Annotations Tool (GREAT) [13]. Published hESC RNA-seq data [9] were used for expression analyses.

### Dot blots

Fully hydroxymethylated DNA was produced by endpoint PCR using Phusion polymerase (NEB, Ipswich, MA, USA) and hm-dCTP (Bioline, Tauton, MA, USA) followed by PCR purification (Qiagen, Valencia, CA, USA). Unmethylated and fully methylated control DNAs were produced in the same manner with dCTP and m-dCTP (NEB, Ipswich, MA, USA), respectively. Various amounts of DNA were denatured, snap cooled and dotted onto positively charged nylon membranes (Roche, Indianapolis, IN, USA). Membranes were crosslinked, blocked with 5% milk, and incubated with Active Motif (1:10,000) 5-hmC antibody for 1 hour. Membranes were washed and then incubated with anti-rabbit secondary horseradish peroxidase-linked antibody (CST, Danvers, MA, USA) for 1 hour, washed, and developed with ECL reagent (CST) and Biomax MS film (Kodak). DNA sequences were (primer sequences in bold):

12 CG-**TACTCTATACTCTACTCATC**ATTACACGCGCGATATCGTTAACGATAATTCGCGCGATTACGATCGATAACGCGTTAATAT**GAGATATGAGATGTGTATG**; 6 CG-**TACTCTATACTCTACTCATC**ATTACAATATATATATCGTTAACGATAATTCGCGCGATTACGATTTATAATTAATTAATAT**GAGATATGAGATGTGTATG**; 3 CG-**TACTCTATACTCTACTCATC**ATTACAATATATATATAATTAATTATAATTCGCGAAATTACGATTTATAATTAATTAATAT**GAGATATGAGATGTGTATG**; 1 CG-**TACTCTATACTCTACTCATC**ATTACAATATATATATAATTAATTATAATTAACGAAATTATAATTTATAATTAATTAATAT**GAGATATGAGATGTGTATG**.

### Validation of hydroxymethylated loci using MspI restriction enzyme and beta-glucosyltransferase

Human stem cell genomic DNA (5 to 10 μg) was treated with the EpiMark 5-hmC and 5-mC Analysis Kit as per the included protocol (NEB). Briefly, DNA was either glucosylated with beta-glucosyltransferase and UDP-Glc or mock treated with beta-glucosyltransferase and no UDP-Glc for 12 to 18 hours. These reactions were then split into three and mock digested, digested with MspI, or with HpaII for at least 4 hours. Samples were treated with proteinase K that was then heat inactivated. All DNA were diluted to a final concentration of 16 ng/μl to be used for PCR analysis. Quantitative PCR was completed with iQ SYBR Green Supermix (Biorad, Hercules, CA, USA) using a CFX384 Real-Time PCR Detection System (Biorad). Primers used for quantitative PCR are listed in Table S1 in Additional file 1.

## Abbreviations

5hmC: 5-hydroxymethylcytosine; 5mC: 5-methylcytosine; bp: base pair; ESC: embryonic stem cell; H3K4me1: histone H3 mono-methylated at lysine 4; H3K27ac: histone H3 acetylated at lysine 27; hESC: human embryonic stem cell; hmeDIP: hydroxymethyl-DNA immunoprecipitation; RPKM: reads per kilobase per million mapped reads; TFBS: transcription factor binding site.

## Authors' contributions

HS, SP and SEJ designed the study. SF and SMK performed the experiments. HS and SEJ analyzed the data. HS wrote the paper.

## Supplementary Material

Additional file 1 **Supplementary figures and table**. Figure S1: 5hmC dot blots on oligos with varying amounts of 5hmC. Figure S2: characterization of defined 5hmC peaks. Figure S3: correlation of 5hmC and gene expression. Figure S4: 5hmC and different classes of putative enhancers [12]. Figure S5: gene ontology analysis of genes that overlap with 5hmC peaks [13]. Figure S6: genome-browser views of 5hmC, enhancers and TFBSs [9,14,15]. Figure S7: sequence composition over 5hmC regions in different genomic locations. Table S1: primers used for quantitative PCR. Click here for file
