# Supplementary material for: 5-Hydroxymethylcytosine is associated with enhancers and gene bodies in human embryonic stem cells
Source: Genome Biol. 2011 Jun 20;12(6):R54. doi: 10.1186/gb-2011-12-6-r54 (PMC3218842; doi:10.1186/gb-2011-12-6-r54)
Supplement: Additional file 1 — Supplementary figures and table. Figure S1: 5hmC dot blots on oligos with varying amounts of 5hmC. Figure S2: characterization of defined 5hmC peaks. Figure S3: correlation of 5hmC and gene expression. Figure S4: 5hmC and different classes of putative enhancers [12]. Figure S5: gene ontology analysis of genes that overlap with 5hmC peaks [13]. Figure S6: genome-browser views of 5hmC, enhancers and TFBSs [9,14,15]. Figure S7: sequence composition over 5hmC regions in different genomic locations. Table S1: primers used for quantitative PCR. [file gb-2011-12-6-r54-S1.PDF]

Supplemental Figure Legends

Figure S1. 5hmC dot blots on oligonucleotides with varying amounts of 5hmC.

Figure S2. Characterization of defined 5hmC peaks.

(a) Distribution of sizes of 5hmC regions. Mean=1571.0bp; median=1249bp; standard deviation=1200.1bp; maximum=25749bp; minimum=249bp.

(b) Distribution of distances between 5hmC regions. Mean=192.56kb; median=45kb; standard deviation=760.48kb; maximum=31468.5kb; minimum=1kb.

(c) Distribution of 5hmC regions in 24 chromosomes.

Figure S3. Correlation of 5hmC and gene expression.

(a) 5hmC over genes. 5hmC peak density was plotted over RefSeq genes in 500 base-pair windows.

(b) Number of RefSeq annotations with given log10(RPKM) values were plotted. "Genes with 5hmC region in body" were genes that contained at least one 5hmC region between the annotated transcription start and termination sites. "Genes with 5hmC region in promoter" were genes that contained at least one 5hmC region between the annotated transcription start and 5kb upstream (i.e.5' end) of the transcription start site.

Figure S4. 5hmC and different classes of putative enhancers.

(a) Percentage of active (Class I) and poised (Class II) enhancers[12] overlapping with 5hmC regions. Random regions with the same number and size distribution as the putative enhancers were generated and overlap with 5hmC regions was calculated 100 times. Error bars represent standard deviation.

(b) Percentage of 5hmC marked enhancers overlapping with Class I and Class II enhancers. Random regions with the same number and size distribution as the 5hmC marked enhancers were generated and overlap with putative enhancers was calculated 100 times. Error bars represent standard deviation.

Figure S5. GO analysis of genes that overlap with 5hmC peaks.

Gene Ontology biological processes analysis of genes that are associated with 5hmC regions using GREAT[13]. The y-axis corresponds to the –log10 of the binomial raw P-values.

Figure S6. Genome-browser views of 5hmC, enhancers and TFBS.

(a) 5hmC over genes specifically expressed in human ES cells[15]. hmeDIP-seq read densities (reads/bp/million uniquely mapping reads) were plotted in 200bp windows. Predicted enhancers[9] and TFBS[14] are also shown.

(b) 5hmC over control genes.

Figure S7. Sequence composition over 5hmC regions in different genomic locations.

(a) Base composition of the Watson strand over the 5' and 3' boundaries of 5hmC regions overlapping with RefSeq genes.

(b) Base composition over 5hmC regions overlapping with predicted hESC enhancers.

(c) Base composition over 5hmC regions neither overlapping with RefSeq genes nor predicted enhancers.

Figure S1

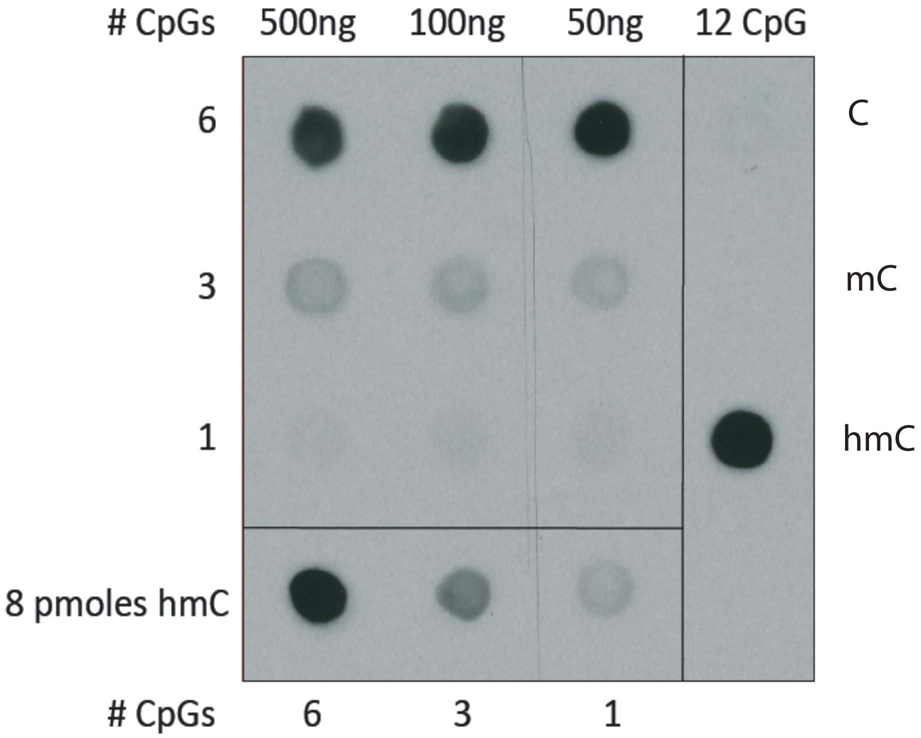

Figure S2

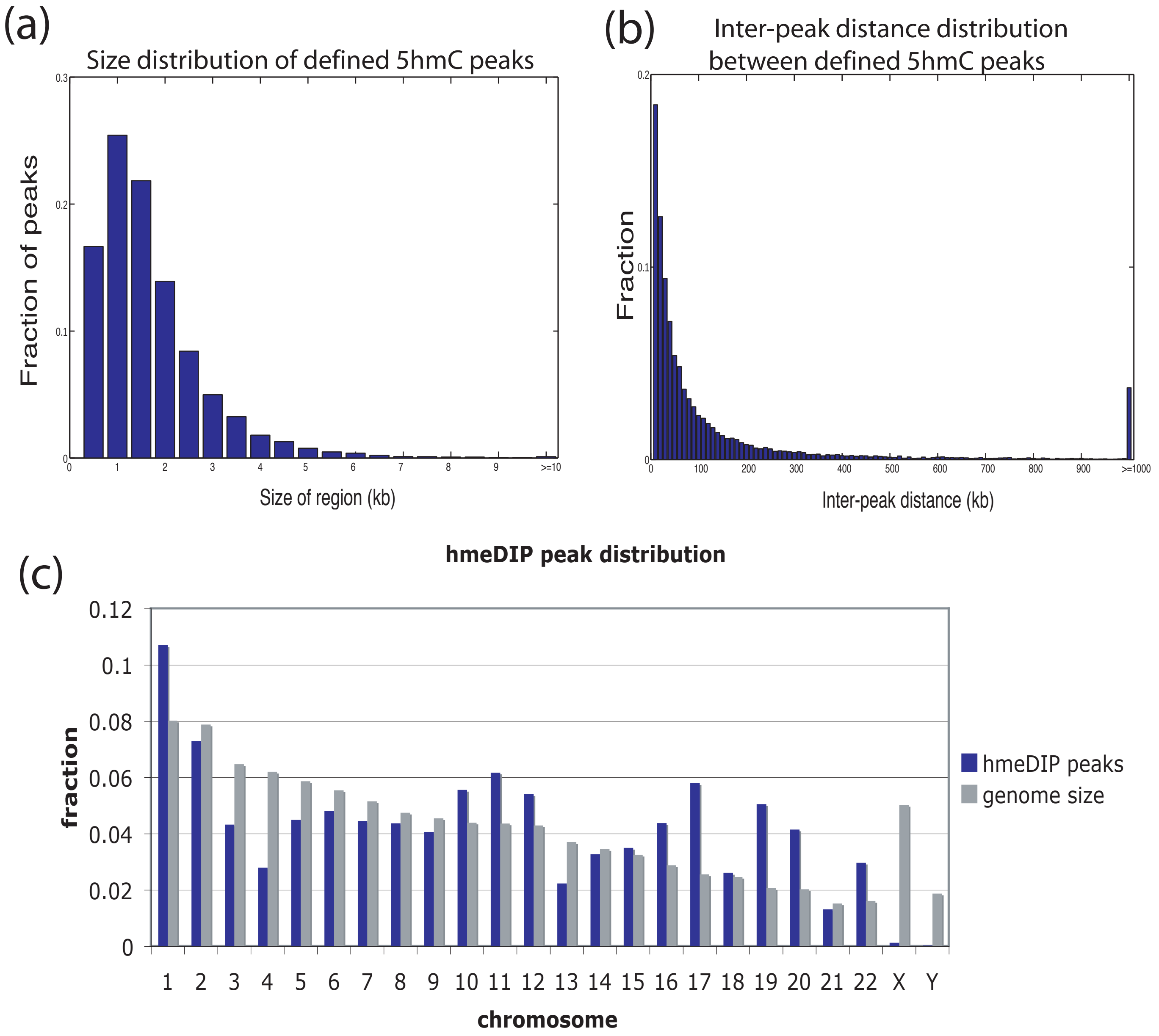

(a)
Size distribution of defined 5hmC peaks
Fraction of peaks
0
0.1
0.2
0.3
0 1 2 3 4 5 6 7 8 9 >=10
Size of region (kb)
(b)
Inter-peak distance distribution between defined 5hmC peaks
Fraction
0
0.1
0.2
0 100 200 300 400 500 600 700 800 900 >=1000
Inter-peak distance (kb)
(c)
hmeDIP peak distribution
fraction
0
0.02
0.04
0.06
0.08
0.1
0.12
1 2 3 4 5 6 7 8 9 10 11 12 13 14 15 16 17 18 19 20 21 22 X Y
chromosome
hmeDIP peaks
genome size

Figure S3

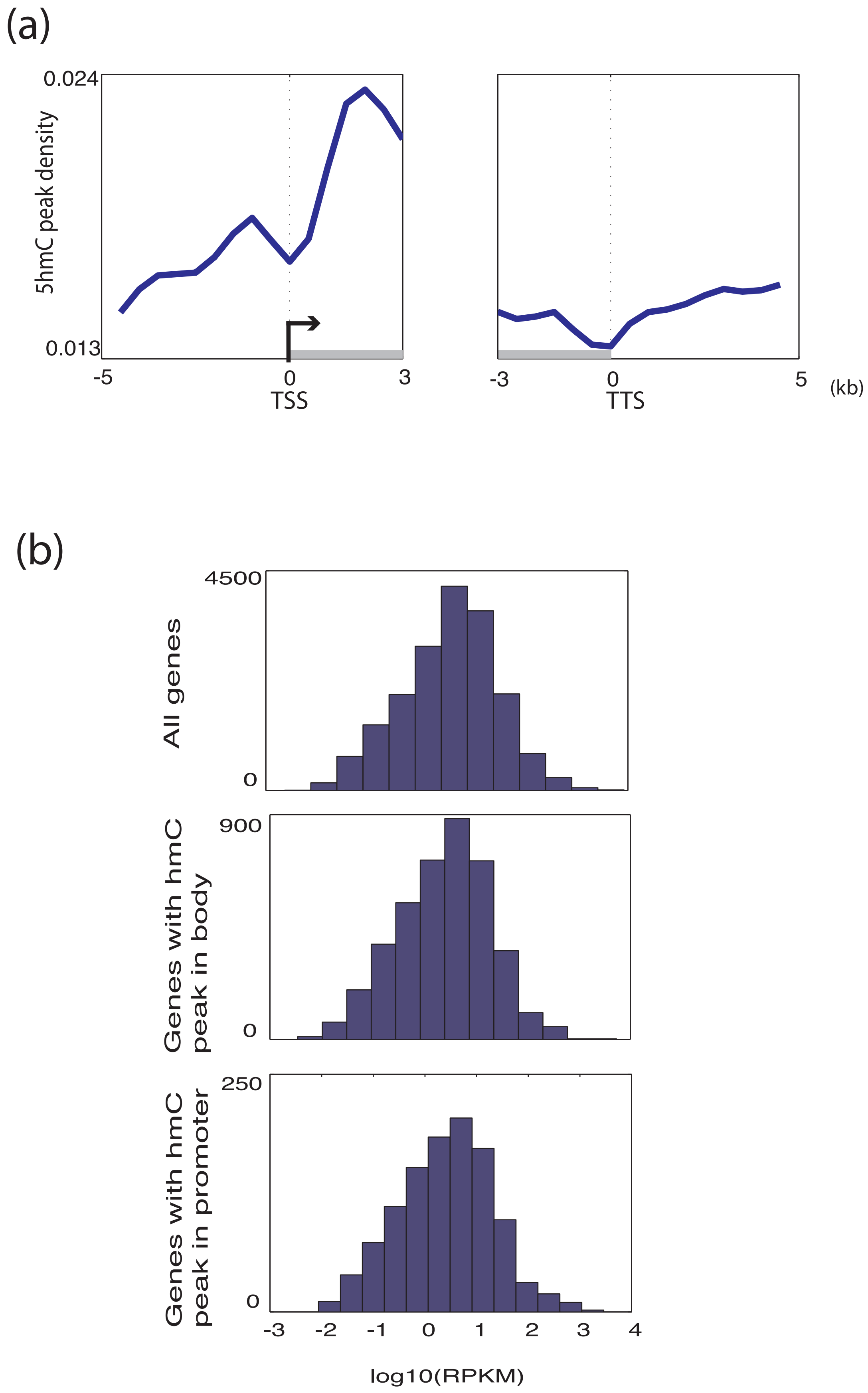
(a)
0.024
0.013
5hmC peak density
-5
0
3
TSS
-3
0
5
TTS
(kb)
(b)
4500
0
All genes
900
0
Genes with hmC peak in body
250
0
Genes with hmC peak in promoter
-3
-2
-1
0
1
2
3
4
log10(RPKM)

Figure S4

(a)

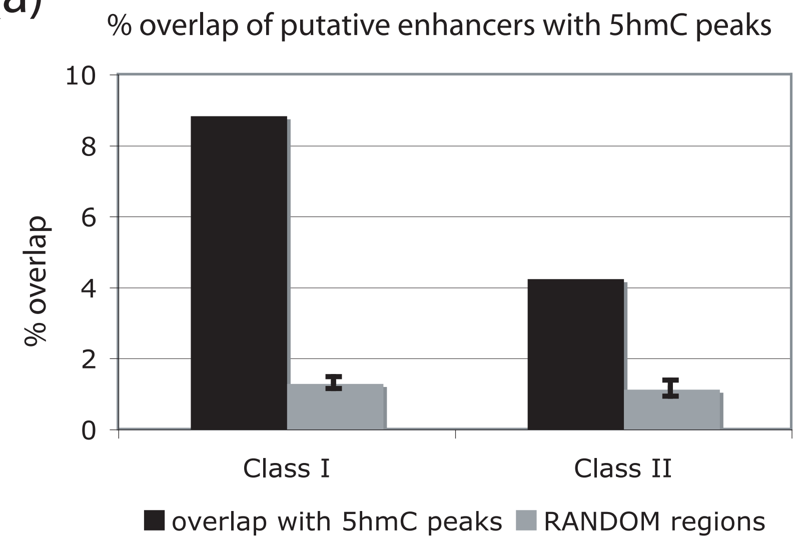
% overlap of putative enhancers with 5hmC peaks
10
8
6
4
2
0
% overlap
Class I
Class II
overlap with 5hmC peaks
RANDOM regions

(b)

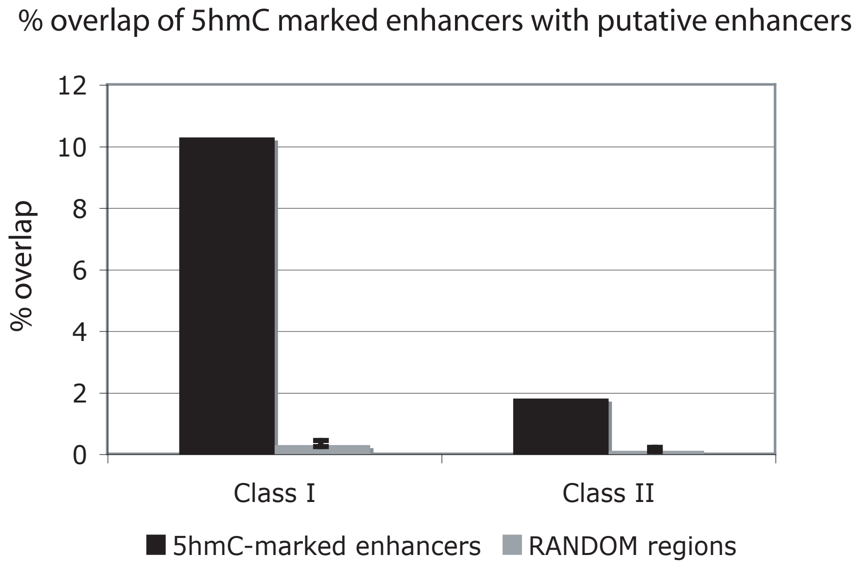
% overlap of 5hmC marked enhancers with putative enhancers
12
10
8
6
4
2
0
% overlap
Class I
Class II
5hmC-marked enhancers
RANDOM regions

Figure S5

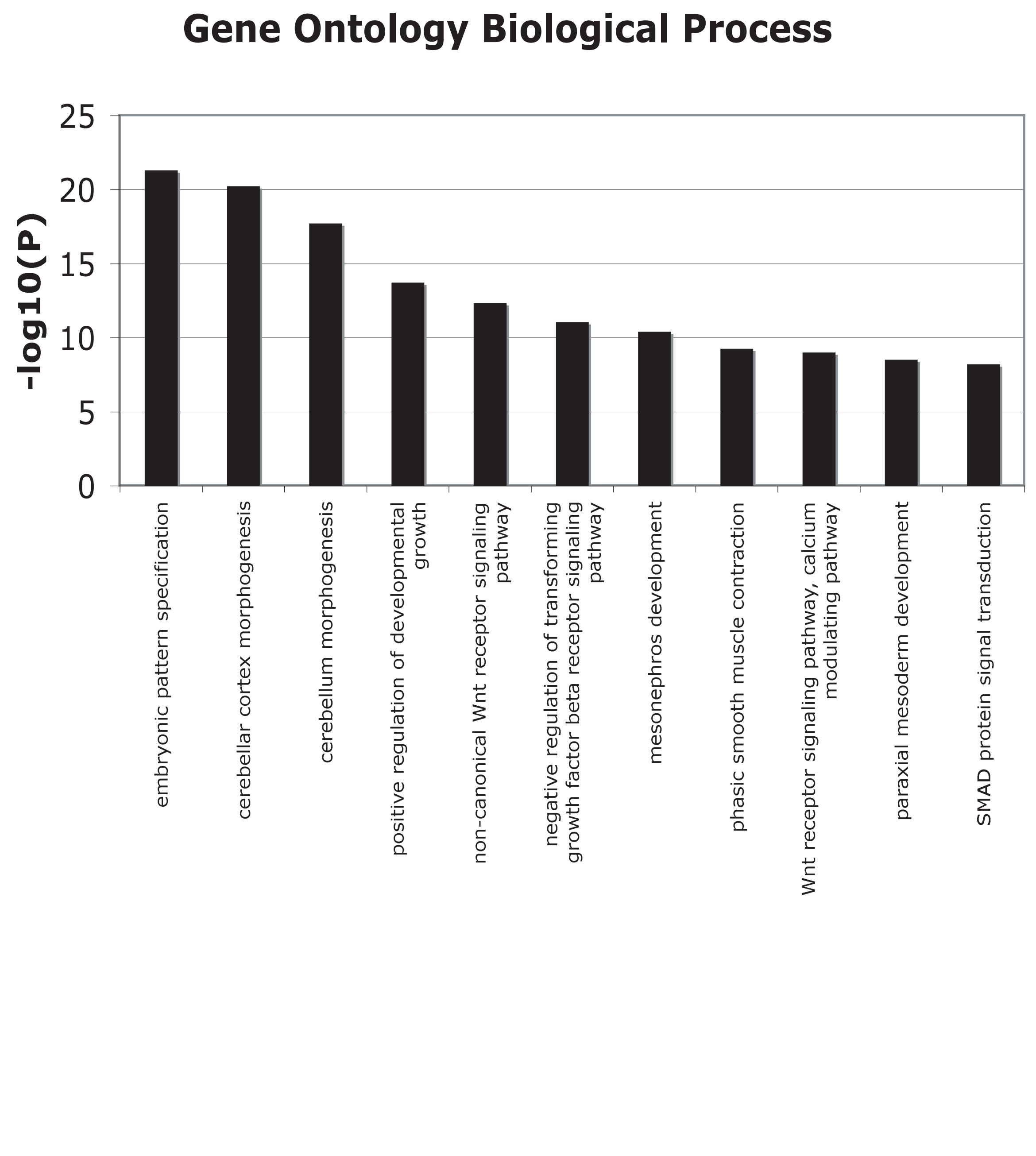

Gene Ontology Biological Process
-log10(P)
25
20
15
10
5
0
embryonic pattern specification
cerebellar cortex morphogenesis
cerebellum morphogenesis
positive regulation of developmental growth
non-canonical Wnt receptor signaling pathway
negative regulation of transforming growth factor beta receptor signaling pathway
mesonephros development
phasic smooth muscle contraction
Wnt receptor signaling pathway, calcium modulating pathway
paraxial mesoderm development
SMAD protein signal transduction

Figure S6

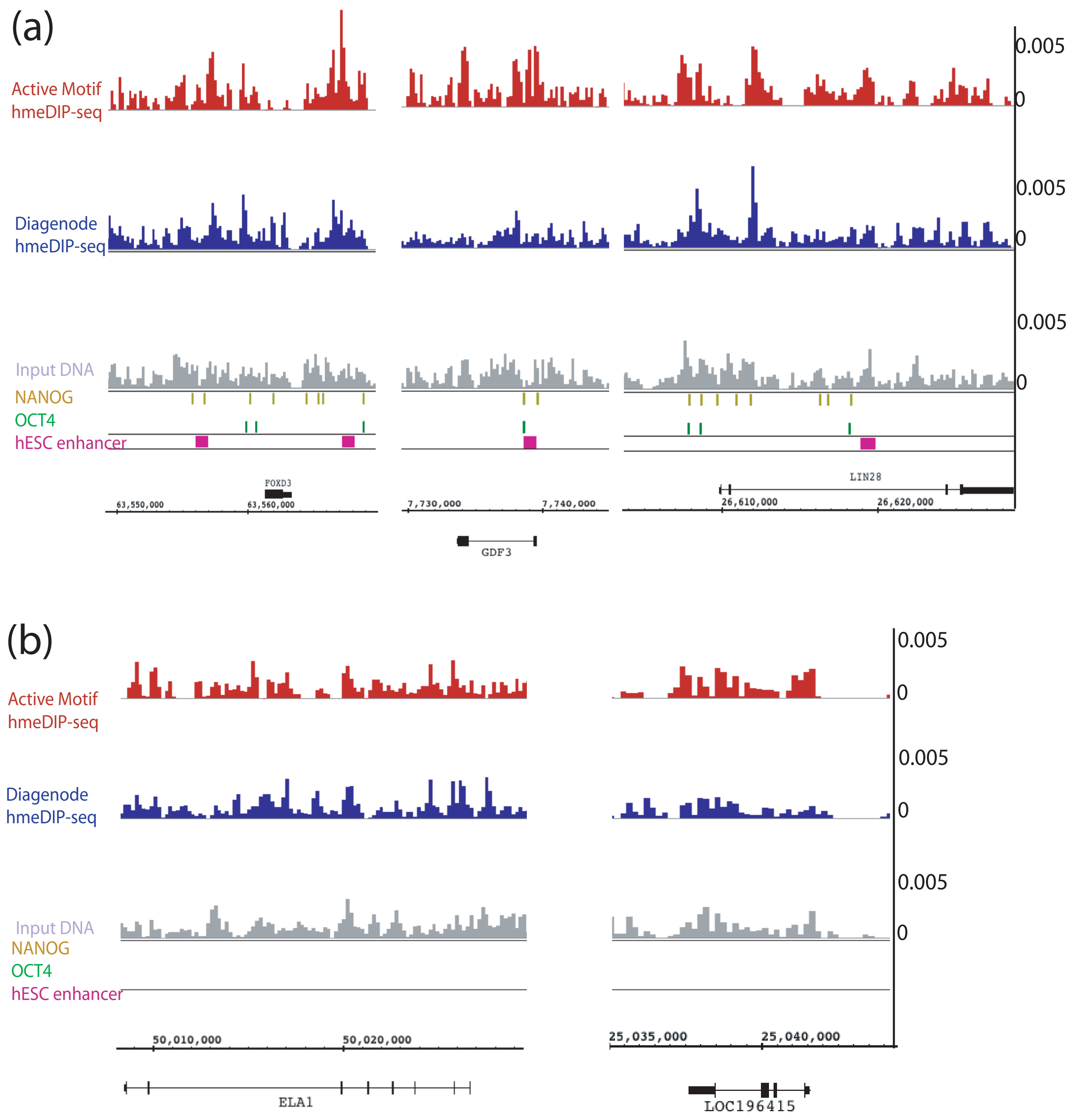
(a)
Active Motif
hmeDIP-seq
Diagenode
hmeDIP-seq
Input DNA
NANOG
OCT4
hESC enhancer
0.005
0
0.005
0
0.005
0
FOXD3
63,550,000
63,560,000
7,730,000
7,740,000
GDF3
LIN28
26,610,000
26,620,000
(b)
Active Motif
hmeDIP-seq
Diagenode
hmeDIP-seq
Input DNA
NANOG
OCT4
hESC enhancer
0.005
0
0.005
0
0.005
0
50,010,000
50,020,000
ELA1
25,035,000
25,040,000
LOC196415

Figure S7

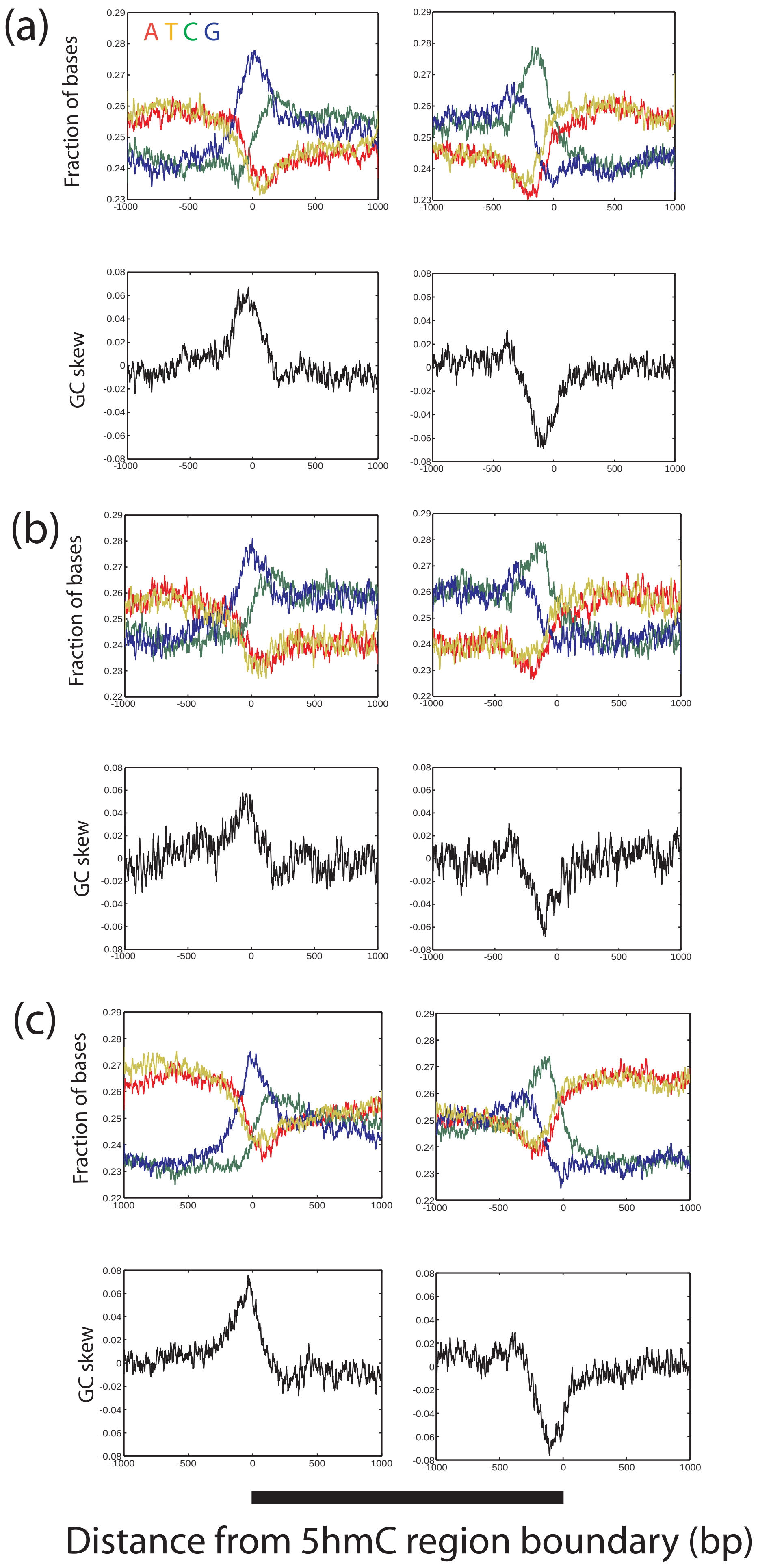
(a)
(b)
(c)
A T C G
Fraction of bases
GC skew
Distance from 5hmC region boundary (bp)

Table S1. Primers used for quantitative PCR.

5hmC enriched regions	Chr1: 11321049	CTCTTCCTCCTCCTCGTCCT
		CATCCAGGAACCACAGTCCT
	Chr1: 149533282	CATCACCTGGAAAGGGAAGA
		CCCTGCAAATTAGGGATGAA
	Chr1: 83930958	ACAGAGAACGGCCACTGACT
		TTGCAGGAGTGAAAGTGTGG
	Chr1: 232733772	GTGGCTGTAGCAGTGGAGGT
		GCTCCTGGTCTTGCTTTGAC
control regions	Chr1: 149536730	CCAAGCCCAGCTAATGTATG
		CGCCTAATCCCAGCTATTTG
	Chr2: 135171597	CCAGCCTGGTTGCTAAAAGT
		TGGTGGCAGGCACCTGTA
